# Signals for Entrepreneurial Family Lending: Psychological Capital as an Intent Signal

**DOI:** 10.3389/fpsyg.2022.797615

**Published:** 2022-02-18

**Authors:** Xue Zhou, Ling Zhang, Xiaoyun Su, Ekaterina Shirshitskaia

**Affiliations:** Business School, Qingdao University, Qingdao, China

**Keywords:** psychological capital, corporate competitive advantage, firm performance, family lending raised, signaling theory

## Abstract

Family financing has become a powerful channel for entrepreneurs to obtain entrepreneurial funding. How do family members use intent and quality signals to select new ventures to provide lending support? Building on the signaling theory, this study provides the first quantitative evidence using a sample of 166 samples of family lenders in China. Our findings reveal that psychological capital can support entrepreneurs to obtain family lending. As an intent signal, psychological capital becomes more influential when quality signals, corporate competitive advantage, and firm performance perform more positively. This study emphasizes that family financing support is not only out of love or altruism and extends the literature concerning the influence of positive psychological capital in financial investment decisions.

## Introduction

Traditional academic literature typically focuses on venture capital or crowdfunding investors. However, they do not specifically focus on family financing, despite serving as the largest single source of startup capital in the world (Steier, 2003). This article will examine lending or investment by the family into an entrepreneurial venture. To begin narrowing the gap between what we know and what we need to know concerning the characteristics of family members investors, this article will examine several key issues. Specifically, we argue that the motivations for family members to provide financial support are included but are not limited to altruism. An expected financial or non-financial return is very likely to exist (Arthurs et al., 2009; Arregle et al., 2015; Lee and Persson, 2016; Sieger and Minola, 2017; Ko and McKelvie, 2018). Accordingly, family members will consider non-business-related and business-related information to make financial decisions. Signaling theory dedicates to reduce information asymmetry between the two parties (Spence, 1978). Moreover, Stiglitz (Stiglitz, 2000) emphasized that reducing the asymmetry of information about quality and information about intent is particularly important. Therefore, family members consider two different kinds of signals. The first kind is an intent signal, which is general information about the entrepreneur’s character and is mainly obtained through a personal relationship with the entrepreneur. The second kind is a quality signal, which is specific information about the entrepreneur’s ability and the quality of the entrepreneurial project. It is mainly obtained through the professional investigation of the entrepreneur and the entrepreneurial project.

Signaling theory points out that quality signals are difficult to imitate, which makes them more valuable to alleviate information asymmetry and support decision-making (Connelly et al., 2011; Colombo, 2021). Additionally, family members do not rely on a single signal to make a judgment; they get information from a signal portfolio, which may contain both costless and costly signals. As Anglin et al. (2018) observed, when there are fewer specific norms of conduct, or when signal receivers are not sophisticated in the current context, the intent signal can be used as a piece of instrument information to help them form the impression or belief of the other party. Positive psychological capital is defined as an individual or organization’s level of psychological resources and consists of four dimensions — hope, optimism, resilience, and confidence (Luthans and Youssef, 2004; Anglin et al., 2018). Therefore, positive psychological capital can enable family members to understand the entrepreneur’s temperament from the above four aspects: hopeful about realizing entrepreneurial dreams, optimistic about entrepreneurial risks, resilience in the face of difficulties, and confidence in their abilities and behaviors. Previous research indicates that entrepreneurs with high positive psychological capital are more trustworthy (Jensen and Luthans, 2006; Anglin et al., 2018). Entrepreneurs do not need to pay obvious costs to have high positive psychological capital in entrepreneurial lending or investment. Still, this intent signal makes family members feel the potential of entrepreneurial projects. Furthermore, compared to analyze an entrepreneurial project from a professional perspective, family members are more likely to know his general temperament information based on his relationship with the entrepreneur. Therefore, we seek to explore the research question: How does the intent signal, positive psychological capital, and quality signal influence the performance of entrepreneurial family lending?

Additionally, we examine how entrepreneurs should convey signals to impact entrepreneurial family lending or investment. Specifically, we seek to determine in two ways: (1) We identify the impact of intent signal on family members’ lending decisions. (2) We investigate the interaction between quality and intent signals on the influence. We probe our research questions by examining how signals sent by entrepreneurs influence entrepreneurial family lending based on the survey of 166 samples in China.

Our work sheds light on several pieces of the literature. First, we contribute to a growing body of evidence, which suggests that entrepreneurial financing decisions are not only driven by quality signals but may also influence intent signals, such as the entrepreneur’s virtue and positive psychological capital (Moss et al., 2015; Anglin et al., 2018). Second, by explicitly focusing on entrepreneurial family financing, we extend the stream of literature concerning the influence of positive psychological capital in financial investment decisions (Anglin et al., 2018). Moreover, whereas this work has typically focused on crowdfunding, we examine entrepreneurial family lending. We find that family members involved in entrepreneur financing are not only out of love or altruism (Lee and Persson, 2016). Third, our findings extend the use of signaling theory within the entrepreneurship literature to include how signal portfolios influence entrepreneurial family financing decisions (Drover et al., 2018). Specifically, we provide evidence for intent signal effect and an interaction effect between intent and quality signal.

## Theoretical Review

### The Entrepreneurial Family Financing Context

Undoubtedly, financial resources are a core resource for startups (Steier, 2003). Due to financial capital from family members having the advantages of low transaction costs, favorite interest, and repayment requirements, family members are considered a relevant available channel of obtaining financial capital for entrepreneurs (Coleman and Robb, 2010). Although scholars believe that getting financial capital from family members is an alternative to traditional financing, such as bank loans because family capital can bring moral burdens and non-financial obligations (Au and Kwan, 2009; Sieger and Minola, 2017). Financial capital from family members dominates entrepreneurial financing (Coleman and Robb, 2010). For example, it found that investigating over 30 countries, capital from close family members—parents, siblings, uncles, and the like—accounts for 42% of informal capital as reported by the surveyed entrepreneurs (Lee and Persson, 2016). Overall, a growing body of research documents that family capital is hailed as the world’s largest single source of startup capital for flexibility and patience (Sieger and Minola, 2017).

The behavior of family members giving entrepreneurs financial capital has been examined from two different views. First, from a family embeddedness perspective, the strong ties between family members and the entrepreneur have been viewed as family embeddedness representing a specific lens of social embeddedness (Aldrich and Cliff, 2003). Family embeddedness indicates frequent interaction and communication between family members and entrepreneurs, which provides entrepreneurs with potential channels for obtaining financial resources (Granovetter, 1973, 1985). Compared to weaker “arm’s length” relations, people engaged in family embeddedness are believed to be long-term generalized reciprocity (Stewart, 2003; Braun and Sieger, 2020). Current studies suggest that different levels of family support (high vs. low) exert mixed influences on entrepreneurial well-being under the context of the daily workload (Xu et al., 2020). Second, from a family value perspective, people are encouraged to cultivate family cohesion, care about the feelings and interests of family members, and maintain dedication, support, and closeness to family members (Freeberg and Stein, 1996; Au and Kwan, 2009). In line with this, family members provide financial support to entrepreneurs to keep family members closely intertwined (Au and Kwan, 2009). However, the existing literature has long recognized that the motivation for family members to provide financial capital to entrepreneurs is not solely from love or kin altruism but rather a fusion of long-term and arm-length relationships (Robb and Robinson, 2014).

### Signaling Theory

Signaling theory provides a solution mechanism to reduce information asymmetry between two parties, such as buyers and sellers (Folger et al., 2021). Spence (1978) interprets the influence of signals on decision-making through the role of education in employment selection and proposes signaling theory. Spence’s analysis of signal theory is mainly from the perspective of the seller (signaler), and Stiglitz (Stiglitz, 1975) proposed the screening theory from the perspective of the buyer (receiver). However, Spence (1976) pointed out that there is no difference between signaling and screening except standpoint, and they are opposite sides of the same coin.

Besides, two core issues discussed around signaling theory are signal information and signal cost. Signaling theory advocates that buyers (receivers) prefer visual and costly information to enrich the understanding of the situation of the seller (signaler) (Connelly et al., 2011). First, the signaling literature has long recognized that the information that can become a signal must be observable (Connelly et al., 2011; Momtaz, 2021). Furthermore, Stiglitz (1975) distinguishes information into general information and specific information and states that “general information is information about characteristics of an individual which affect his productivity in a wide variety of jobs; specific information concerns characteristics which affect his productivity in a specific firm.” Second, the view that the buyer (receiver) favors costly signals has been challenged regarding signal cost. Costly signals, such as educational background, executive experience technical experience, and enterprise-quality have positive effects on entrepreneurs’ access to capital (Gomulya et al., 2019; Epure and Guasch, 2020). Scholars have examined that entrepreneurs’ virtual and positive psychological capital, although costless for entrepreneurs, also plays an essential role in the decisions of investors and other receivers (Zhou et al., 2021). Scholars’ conclusions on costless signals echo the value of general information proposed by Stiglitz (1975). Also, from the perspective of signal cost analysis, Spence (1976) adheres to the usual style of measuring the cost of signal from the perspective of signalers, and existing studies have followed its evaluation method. In addition, Stiglitz (1975) estimates the cost of its screening signal from the view of receivers, and no analysis based on this has been found in previous research. Therefore, this study combines the dual perspectives of signaling and screening to weigh the cost of signal and analyzes the signaling mechanism under the context of entrepreneurial family financing. Combining signal information and signal cost, we found that the quality signal and intent signal proposed by Stiglitz (2000) based on information correspond to the costly signal and cost less signal divided by cost.

**FIGURE 1:**
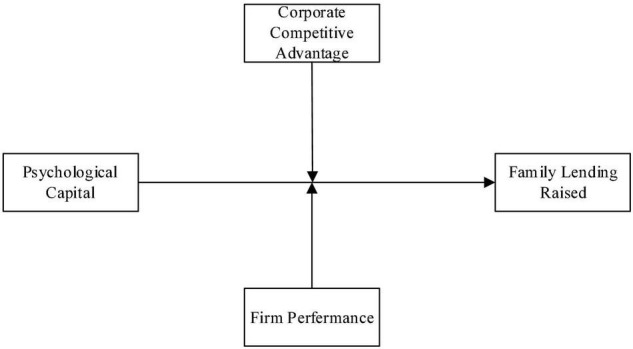
Conceptual model.

In the following sections, we draw from signaling theory to develop a model in which the quality and intent signals are argued to generate positive affection on family members who screen the entrepreneur and subsequently influence their funding decisions. We take positive psychological capital as the intent signal independent variable in our conceptual model and two factors: corporate competitive advantage and firm performance as quality signals independent variables. We examine the signaling mechanism and identify the signal portfolio’s contracture, which influences the lending of family members. Moreover, we further hypothesize that a quality signal will moderate the degree to which an intent signal generates the likelihood of entrepreneurial family lending. Our full conceptual model is depicted in Figure 1.

## Hypothesis Development

### Positive Psychological Capital as Intent Signal

Traditional signal theory points out that easy to observe and difficult to imitate are the two elements of a signal: a quality signal (Spence, 1978, 2002; Connelly et al., 2011). Anglin et al. (2018) believe that the intent signal is also valuable in asymmetric information environments, and use positive psychological capital as an intent signal. They further discovered that positive psychological capital has a positive relationship with crowdfunding performance (Anglin et al., 2018). Positive psychological capital is defined as the level of personal or organizational psychological resources in the four aspects of hope, optimism, resilience, and confidence (Avey et al., 2011; Friend et al., 2016). Considering the crucial role of entrepreneurs in startups, we focus on the positive psychological capital of entrepreneurs (Ensley et al., 2006). The entrepreneur has a high positive psychological capital, as a signal transmits positive information, such as authentic, which will guide investors to tend to optimistically judge entrepreneurial projects (Jensen and Luthans, 2006). Specifically, hope means that the entrepreneur believes that the entrepreneurial goal can be achieved; optimism means that he has positive expectations for entrepreneurial activities; resilience means that he can recover from entrepreneurial setbacks or become stronger; confidence means that the entrepreneur believes that he can complete entrepreneurial activities (Avey et al., 2011).

### Quality Signals

We select quality signals that are closely related to the quality of the entrepreneurial project. Specifically, the quality signals include corporate competitive advantage and firm performance. Prior founding experience supports that these signals influence obtaining investment in the context of venture capital or crowdfunding due to improved information asymmetry (Gerrit et al., 2015; Ko and McKelvie, 2018; Kleinert et al., 2021). In this regard, we argue that these signals still are valuable predictors of entrepreneurial potential in entrepreneurial family financing.

### Intent and Quality Signals

According to Spence (1976), we refer to the quality and intent signals both as signaling, from the entrepreneurs’ standpoint and screening from family members’ standpoint, and a fusion of signal information and signal cost.

For entrepreneurs, the intent signal is endogenous, and the personality or character can be obtained without cost. The quality signal is exogenous, and it is the advantage and performance that need to be spent on company operation and other aspects (Spence, 1976).

For family members, according to the distinction between general information and specific information portrayed by Stiglitz (1975), intent and quality are mainly distinguished by whether they need to spend additional screen costs to be collected. For family members, the intent signal is general information, which can be known through the personal relationship with the entrepreneur. Whether or not it involves a financial interaction with the entrepreneur, the family member will screen this information. Interaction with entrepreneurs does not drive them to spend extra screen costs to obtain such information. However, the quality signal is specific information about the entrepreneur or the entrepreneurial venture, collected based on the professional relationship with the entrepreneur (Stiglitz, 2000). The motivation to obtain this information is triggered by the fact that family members need to be involved in the entrepreneurial venture and take corresponding risks. In this view, they are encouraged to screen-related specific professional information with extra cost (Spence, 1976).

### Signals for Entrepreneurial Family Lending

For family members, the expectation of entrepreneurial family lending is from the perspective of creditors. There is a desire not to bear the risk of entrepreneurship. In other words, there is no entrepreneurial risk-shifting between the entrepreneur and family members. If we study in-depth, there are two different situations of entrepreneurial family lending according to the other requirements for the power of entrepreneurs’ debt commitments.

The first type has low requirements for the strength of entrepreneurs’ debt commitments. Family members do not require entrepreneurs to make firm commitments to the debt. In this case, if family members have positive expectations for entrepreneurial activities, they are willing to provide loans to enable entrepreneurs to obtain benefits. Suppose family members have negative expectations for entrepreneurial activities. The original intention of giving loans is not optimistic about the business. Still, the entrepreneur insists on starting the company, and the family is willing to support him, even if it loses money (Sieger and Minola, 2017). Therefore, in this case, regardless of whether family members have positive or negative expectations for entrepreneurial activities, providing loans to entrepreneurs is out of altruism. The second type has high requirements for the strength of entrepreneurs’ debt commitments. Family members require entrepreneurs to make firm commitments to the debt. At this time, family members provide loans out of positive expectations for entrepreneurial activities, and loans are not allowed to become bad debts. At this time, family members choose to borrow rather than invest because of their attitude toward risk.

In general, in low-debt commitment loans, the decision-making process of family members is more emotional than rational. They provide that loan is out of altruism rather than screening signals. In high debt commitment loans, the decision-making process of family members is more reasonable than perceptual. They will filter signals to consider the core issue of the entrepreneur’s ability to fulfill the debt contract—moral hazard—and attach more importance to the moral quality of entrepreneurs in the relevant signals sent by entrepreneurs (Jensen and Meckling, 1976; Holmstrom and Tirole, 1997). For family members, entrepreneurs’ poor moral qualities can bring default risk. In this situation, the general character information of the entrepreneur is valuable because it can signal to family members whether the entrepreneur has a moral hazard. Therefore, positive psychological capital as the intent signal has a beneficial impact (Stiglitz, 1975; Spence, 1976). Positive psychological capital is defined as an individual or organization’s level of psychological resources and consists of four dimensions—hope, optimism, resilience, and confidence (Anglin et al., 2018). Entrepreneur in high positive psychological capital signals good quality regarding allocating capabilities, resolving risks, achieving goals, and promoting entrepreneurial sustainability. Under the premise that the entrepreneur has a good character, based on the low monitoring costs among family members, the opportunistic behavior of the entrepreneur can be effectively avoided, thereby reducing the risk of debt repayment (Karra et al., 2006). The signaling of positive psychological capital portrays that the entrepreneur is authentic, which leads to a favorable evaluation of repayment credit (Jensen and Luthans, 2006). Accordingly, we hypothesize the following:

Hypothesis 1: Psychological capital, as an intent signal, is positively associated with the likelihood of receiving the entrepreneurial family loan.

### The Moderating Influence of Quality Signals

Although there is a loan relationship between family members and entrepreneurs, there is no direct entrepreneurial risk-shifting. Whether a startup enterprise has good operating performance will still have an impact on family members’ repayment. The excellent development of entrepreneurial enterprises means that entrepreneurs can accumulate more wealth. Conversely, bumping into obstacles everywhere will increase entrepreneurs’ economic and mental pressure. Therefore, for family members, even if it is observed that entrepreneurs’ high debt commitments can provide subjective guarantees for their repayment of borrowings, good business operations can still help provide objective conditions for entrepreneurs to perform their contracts. As specific information about the entrepreneur and entrepreneurial venture, quality signals are beneficial under this condition. Entrepreneurs convey multiple simultaneous signals to family members, which help the family to judge the quality of startups (Bergh et al., 2014; Steigenberger and Wilhelm, 2018). We pay attention to quality calls that can directly reflect the operating conditions of startups, including the corporate competitive advantage and the firm performance. The presence of quality signals can convey professional and specific information which may be used to evaluate the risk of an entrepreneurial venture. Displays of existing achievements of the entrepreneur and the startup lead to more positive evaluations of the entrepreneurial potential by family members. As such, the positive quality signals make the family members prefer to become optimistic and confident that the entrepreneur can successfully pursue his entrepreneurial goal. Accordingly, we hypothesize the following:

Hypothesis 2a: Corporate competitive advantage moderates the relationship between positive psychological capital language and family lending such that increases in corporate competitive advantage strengthen the relationship between positive psychological capital and family lending amount.

Hypothesis 2b: Firm performance moderates the relationship between positive psychological capital language and family lending such that increases in firm performance strengthen the relationship between positive psychological capital and family lending amount.

## Research Design

### Sample Selection and Data Collection

Family members lend to support entrepreneurs is a process by which family members make decisions based on the relevant signals they receive. Therefore, we collect questionnaires from the perspective of investors, that is, family members. Data were collected from March to August 2021. Samples mainly came from the eastern entrepreneurial region of China, where are economically developed and active in entrepreneurship. We collect questionnaires through both online and offline channels. On the one hand, the online channel is to share and collect electronic questionnaires through entrepreneurs to family members who have provided loans to the entrepreneur within 3 years. The specific method of questionnaire collection was as follows: researchers visited the makerspace in the eastern entrepreneurial region of China and conducted interviews with entrepreneurs. After we learnt about the financing situation of entrepreneurs, the entrepreneurs who have obtained family lending in the past 3 years were requested to share the electronic questionnaires link on the Web survey platform (Questionnaire Star) to their family lenders. Therefore, researchers can collect the results online. On the other hand, we have printed paper questionnaires and issued them to family members who have borrowed to support family entrepreneurs in the past 3 years. In addition, we set a question “have you filled in online and offline questionnaires at once” in the questionnaire, to avoid the questionnaire being filled out multiple times by the same person. The content of the questionnaires used by both online and offline is the same. The purpose of using the electronic version is to facilitate the sharing and recycling of the questionnaires. According to the standards of global entrepreneurship observation, we require that startups be established no more than 5 years when they obtain their loan support. At the same time, we stated that family members refer to immediate relatives and three-generation collateral relatives in the questionnaire. Additionally, it is required to fill out the questionnaire according to the circumstances when the entrepreneurs were given loan support.

We used two methods to minimize general method deviation. The first was by implementing anonymous filling methods to reduce the responsibilities of the person filling the questionnaire. The second was to avoid the questionnaire being filled out multiple times by the same person (filling in online and offline questionnaires at once). We sent 178 online questionnaires on Questionnaire Star (a Chinese internet platform) and 72 offline questionnaires. Ultimately, this survey returned 216 questionnaires and excluded incomplete or overlapping questionnaires. We only keep the family lending experience data that occurred before the epidemic. Additionally, we deleted the sample data from the parents of entrepreneurs. We reviewed the quality of the questionnaires and finally saved 166 questionnaires with an effective recovery rate of 76.9%. Among the test participants, 35.5% were women. The educational background distribution was only 9.6% for college and below, 18.7% for master’s degree students, and upper. As the research object includes entrepreneurs, the subjects’ ages were mainly distributed between 20–30 years old and 30–40 years old, accounting for 53.6 and 42.2%, respectively.

### Variable Measurement

Translation and backtranslation processes were adopted for the adopted foreign scales to avoid semantic deviation. This study’s measurement of variables uses a seven-point Likert scale, with 1 indicating complete disagreement and 7 indicating full compliance. The necessary information of the enterprise is measured using the form of selection or filling in the blanks.

#### Psychological Capital

Drawing on the research results of Luthans and Youssef (2004), 24 items were used for measuring millennials’ entrepreneurial values, which include hope, optimism, resilience, and confidence. The internal consistency of Cronbach’s α coefficient was 0.887.

#### Corporate Competitive Advantage

Learning from Schulte (Schulte, 1999), six items were used to measure corporate competitive advantage, which include the followings. (1) The company can provide the customers with products or services at a lower cost. (2) The company can provide customers with multifunctional, high-performance products or services. (3) The company can execute operating procedures more quickly and more efficiently. (4) The company can flexibly adapt to rapid changes and market response faster than competitors. (5) The company pays more attention to customer needs. (6) The company’s market share is multiplying. The internal consistency of Cronbach’s α coefficient was 0.691.

#### Firm Performance

Refer to the questionnaire used by Anderson et al. (2009) to measure the performance of new ventures from the two dimensions of growth and profitability. The measurement scale contains a total of eight items, and there are five growth measurement items, including items, such as “compared with other startups, the growth rate of the company’s market share.” There are three profitability measurement items, including items, such as “compared with other startups, the company’s market share.” The internal consistency of Cronbach’s α coefficient was 0.846.

#### Family Lending Raised

The number of loans is reflected in the four ranges, below 50,000, 50,000 to 100,000, 100,000 to 200,000, and more than 200,000 yuan.

#### Control Variables

According to previous related studies, the personal characteristics of entrepreneurs and the characteristics of enterprises will have an impact on the financing of startups. Existing research methods, age, gender, educational background, number of startups, previous industry experience, firm age, and firm size are used as a control variable.

## Empirical Analysis and Results

### Convergent Validity

In this article, SPSS and AMOS are used for statistical analysis. Convergent validity was a measure of the model fit. The average variance extracted (AVE) showed the degree of correlation between the construct and its indices, with a greater fit achieved with a stronger correlation. Any composite-reliability (CR) rating higher than 0.7 (Hair et al., 2017) suggests that the construct was internally acceptable. In this study, the AVE of all variables was higher than 0.5, and the CR of all variables was higher than 0.7 (Table 1).

**TABLE 1:** Indicators of measurement.

Variable	Items	Factor loading	Average variance extracted (AVE)	Composite reliability (CR)	Cranach’s alpha
Psychological capital	Confidence1	0.792	0.533	0.964	0.887
	Confidence2	0.606			
	Confidence3	0.756			
	Confidence4	0.572			
	Confidence5	0.804			
	Confidence6	0.659			
	Hope1	0.749			
	Hope2	0.627			
	Hope3	0.637			
	Hope4	0.773			
	Hope5	0.560			
	Hope6	0.803			
	Resilience1	0.772			
	Resilience2	0.864			
	Resilience3	0.742			
	Resilience4	0.738			
	Resilience5	0.563			
	Resilience6	0.623			
	Optimism1	0.769			
	Optimism2	0.903			
	Optimism3	0.901			
	Optimism4	0.519			
	Optimism5	0.752			
	Optimism6	0.845			
Corporate competitive advantage	CCA1	0.893	0.616	0.902	0.691
	CCA2	0.566			
	CCA3	0.900			
	CCA4	0.874			
	CCA5	0.853			
	CCA6	0.526			
Firm performance	Growth1	0.592	0.593	0.919	0.846
	Growth2	0.706			
	Growth3	0.693			
	Growth4	0.831			
	Growth5	0.683			
	Profitability1	0.849			
	Profitability2	0.878			
	Profitability3	0.876			

### Discriminant Validity

Discriminant validity is the extent to which a construct is truly distinct from other constructs by empirical standards (Hair et al., 2017). To test the discriminant validity of the variables involved in this article, confirmatory factor analysis of psychological capital, corporate competitive advantage, firm performance, and family lending was raised. The AMOS confirmatory factor analysis results are shown in Table 2. The data fit of the four-factor model (χ2/df = 1.56, RMSEA = 0.05; SRMR = 0.05; CFI = 0.92; TLI = 0.91) is the most ideal, which is significantly better than other models. It shows that the four variables involved in this article have good discriminant validity.

**TABLE 2:** Confirmatory factor analysis results.

Models	χ 2	df	χ 2/df	RMSEA	SRMR	CFI	TLI
Four factors	168.49	108	1.56	0.05	0.05	0.92	0.91
Three factors a	204.97	122	1.68	0.06	0.06	0.87	0.85
Two factors b	274.77	129	2.13	0.08	0.07	0.82	0.81
One factor c	332.76	141	2.36	0.10	0.08	0.79	0.77

*a, psychological capital + family lending raised, corporate competitive advantage, firm performance; b, psychological capital + family lending raised + corporate competitive advantage, firm performance; c, psychological capital + family lending raised + corporate competitive advantage + firm performance.*

### Descriptive Statistical Analysis

Descriptive statistics mainly display each variable’s average value, standard deviation, and correlation coefficient (as shown in Table 3). Existing research methods, age, gender, educational background, number of startups, previous industry experience, firm age, and firm size are used as a control variable. According to the results of correlation analysis, psychological capital is significantly positively correlated with family lending raised (*r* = 0.16, *p* < 0.05), corporate competitive advantage and family lending raised (*r* = 0.24, *p* < 0.01), firm performance and family lending raised (*r* = 0.18, *p* < 0.05) all showed a significant positive correlation. It provides specific support for the subsequent hypothesis argumentation in this article.

**TABLE 3:** Descriptive statistical analysis.

Variable	Mean	SD	1	2	3	4	5	6	7	8	9	10
Family lending raised	2.33	1.02										
Psychological capital	5.32	0.70	0.16*									
Corporate competitive advantage	5.12	0.81	0.24**	0.63**								
Firm performance	4.59	0.92	0.18*	0.42**	0.62**							
Age	2.49	0.58	0.04	0.25**	0.08	0.07						
Gender	1.36	0.48	–0.02	–0.07	–0.02	0.10	0.09					
Educational background	4.08	0.68	–0.10	0.01	–0.04	0.06	0.09	0.06				
Number of startups	1.50	0.72	0.05	0.13	0.19*	0.07	0.05	0.01	–0.11			
Previous industry experience	5.83	4.17	0.13	0.24**	0.09	0.09	0.50**	0.00	0.00	0.05		
Firm Age	2.95	1.30	0.05	0.10	0.10	0.23**	0.08	0.04	0.09	0.16*	0.16*	
Firm Size	3.36	1.67	0.04	–0.01	0.14	0.25**	0.08	0.17*	0.14	0.18*	0.15	0.46**

*Significantly correlated at the 0.05 level (bilateral).

**Significantly correlated at the 0.01 level (bilateral).

### Common Method Bias

Common method bias often arises when the questionnaire method is used for data collection. This questionnaire adopts an anonymous evaluation method; however, common method bias for the same participants remains unavoidable. We used Harman’s single factor test to perform an unrotated factor analysis on all collected questionnaire item data to test the common method bias. The variance explained by the first principal component is 20.00%. It does not constitute half of the variance explained by the total variable (66.88%). Therefore, the standard method bias of the sample data was within an acceptable range.

### Hypothesis Testing

To avoid the influence of multicollinearity on the regression results, a multigroup hierarchical regression method is used for analysis. The average value of multiple items is used as the variable score for variables with various measurement items, and the main variables are centrally processed. The regression analysis results of the hypothesis test are shown in Table 4. The VIF values of all variables in each model are below two, which indicate no severe collinearity problem. The models in Table 4 all analyze the entrepreneur’s age, gender, educational background, number of startups, previous industry experience, firm age, and firm size as control variables. Whereas psychological capital significantly positively affects family lending raised (β = 0.15, *p* < 0.01), H1 is supported; whereas corporate competitive advantage significantly positively regulates the relationship between psychological capital and family lending raised (β = 0.17, *p* < 0.01), H2a is supported; whereas firm performance significantly positively regulates psychological capital and family, the relationship of lending raised (β = 0.19, *p* < 0.01), H2b is supported.

**TABLE 4:** Psychological capital and family lending raised.

	Model 0	Model 1	Model 3	Model 4
	
Variables	Controls	Main effect	Corporate competitive advantage moderators	Firm performance moderators
	
	1	2	3	4
Age	−0.02 (−0.26)	−0.05 (−0.53)	−0.03 (−0.36)	−0.05 (−0.56)
Gender	−0.01 (−0.13)	0.00 (0.00)	−0.03 (−0.38)	−0.03 (−0.41)
Educational background	−0.10 (−1.24)	−0.10 (−1.27)	−0.07 (−0.83)	−0.10 (−1.31)
Number of startups	0.02 (0.31)	0.01 (0.09)	−0.03 (−0.37)	0.03 (0.36)
Previous industry experience	0.13 (1.44)	0.11 (1.20)	0.10 (1.08)	0.09 (1.08)
Firm age	0.03 (0.38)	0.02 (0.22)	0.04 (0.48)	0.02 (0.24)
Firm size	0.01 (0.14)	0.03 (0.30)	−0.03 (−0.33)	−0.04 (−0.43)
Psychological capital		0.15** (1.79)	0.01* (0.10)	0.10 (1.10)
Corporate competitive advantage			0.24 (2.36)	
Firm performance				0.11 (1.17)
Psy Cap × Cor Com Adv			0.17** (2.16)	
Psy Cap × Firm Per				0.19** (2.29)
VIF maximum	1.39	1.42	1.88	1.48
R square	0.03	0.06	0.11	0.10
Δ R square	0.03	0.03	0.05	0.04

N = 166, ** and * indicate p < 0.01 and p < 0.05, respectively.

**FIGURE 2:**
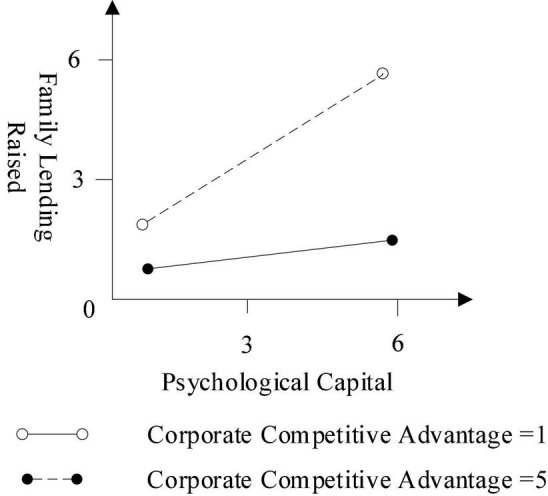
The moderator of corporate competitive advantage.

**FIGURE 3:**
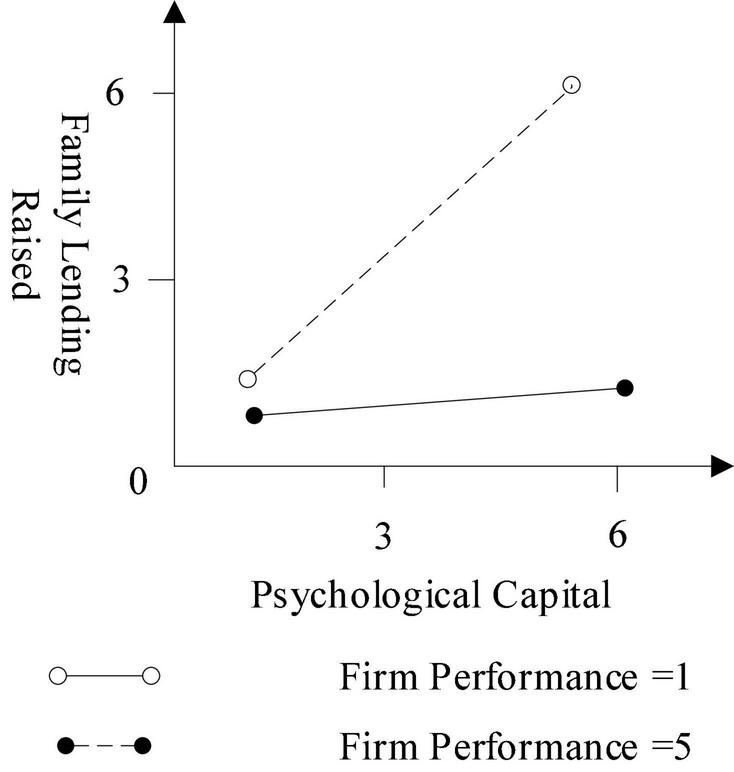
The moderator of firm performance.

Regarding the psychological capital related to corporate competitive advantage and firm performance, Models 3 and 4 (in combination with Figures 2, 3) reveal that the intent signal becomes more influential when corporate competitive advantage and firm performance are better. Supporting Hypotheses 2a and 2b, both interaction terms are positive and statistically significant.

## Discussion

### Theoretical Implications

This study makes three contributions to family financing and entrepreneurial finance research in particular and entrepreneurship signaling research in general.

First, we provide a new analytical perspective for the family financial investment decisions. Previous research on family financing focuses on the theoretical mechanism of family financial decision from family embeddedness perspective or family value perspective (Au and Kwan, 2009; Braun and Sieger, 2020; Liao, 2021), but we emphasize that family financing support is not only out of love or altruism and signals are influential. Our data indicated that signals play an important role when family members value the entrepreneur’s commitment to fulfilling the contract.

Second, our study expands entrepreneurial finance research, especially entrepreneurship finance signaling research, whereas previous work has typically focused on crowdfunding context and venture investment (Anglin et al., 2018; Colombo et al., 2019). We figure out that the information of entrepreneurs and enterprise can affect the family lending raised as signals based on signaling theory. Family members can obtain the positive psychological capital of the entrepreneur through the common communication with the entrepreneur. Our study broadens the cognition of the effect of the intent signal and quality signal and also is conducive to a complete characterization of the influence process of family financial investment decisions.

Third, we contribute to a growing body of evidence, which suggests that entrepreneurial financing decisions are not only driven by quality signals but may also be influenced by intent signals, such as the entrepreneur’s virtue and positive psychological capital. By explicitly focusing on entrepreneurial family financing, we extend the stream of literature concerning the influence of positive psychological capital in financial investment decisions (Anglin et al., 2018). This study extends the use of signaling theory within the entrepreneurship literature to include how signal portfolios influence entrepreneurial family financing decisions (Drover et al., 2018). Our results reveal that intent signal, psychological capital, becomes more influential when quality signals, corporate competitive advantage, and firm performance perform more positively.

### Practical Implications

Entrepreneurs who hope to obtain financial support from family members must first clarify the strength of the relationship between family members and themselves. Very close relatives, such as parents, are willing to support entrepreneurs’ career development out of love. In helping entrepreneurs, they will worry more about the impact of entrepreneurial projects on entrepreneurs’ bodies and minds rather than worry about whether entrepreneurs can repay the loan on time. Relatives who are relatively distant from each other will rationally evaluate the entrepreneur’s debt repayment commitment and contract performance when deciding whether to provide financial support to entrepreneurs. Our data show that psychological capital, as a manifestation of the reliability of entrepreneurs, is a crucial signal that directly affects the economic decisions of family members. Therefore, entrepreneurs should show positive psychological capital in getting along with their relatives and offer their relatives the characteristics of self-confidence, optimism, and tenacity. At the same time, this study found that good performance and competitive advantages of entrepreneurial companies can encourage relatives to form positive judgments about the development of the company, thereby enhancing the perception of timely payment and thus more willing to provide financial support to entrepreneurs. Therefore, to obtain loans from relatives, entrepreneurs can use optimistic language to describe the company’s market prospects, competitive industry position, etc., and even make appropriate decorations under the premise of controllable risks.

### Shortcomings and Prospects

In addition to the research implications of our findings, the study’s limit may open opportunities for further research. First, a limitation pertains to our research context. This research focused on family lending. However, entrepreneurial family financial support also includes family investment. Additional research can try to signal family investment. Compared to family lending, the expectation of entrepreneurial family investment is from the investor’s perspective. The risk of entrepreneurship needs to be assumed by the family, and the success of entrepreneurship is closely related to capital repayment. Simply, there is entrepreneurial risk-shifting between the entrepreneur and family members. In entrepreneurial family investment, family members will decide after examining the risk associated with the venture based on several signals.

Second, though we collect data from family lenders and delete the sample if the family lenders are parents of entrepreneurs, it still cannot be ruled out that our model may contain situations that support entrepreneurs out of love. In this case, family members are willing to provide loans to enable entrepreneurs to obtain benefits. The original intention of giving loans is to support entrepreneurs insisting on starting the business. The existence of such a sample will weaken the influence of the signal we are studying.

Third, further research might expand our study regarding its theoretical scope. We focused on intent signal, psychological capital, and two quality signals, corporate competitive advantage and firm performance. Yet, a variety of additional signals also might be relevant for the family financial decision. For example, entrepreneurial passion and other soft skills by entrepreneurs, social capital (Khoury et al., 2013), or language signals conveyed on the pitch decks (Anglin et al., 2018) are relevant for other entrepreneurial finance players. Additionally, family members do not rely on a single signal to make a judgment. They get information from the signal portfolio (Drover et al., 2018), which may contain both intent and signal signals. Continued research might identify the signal portfolio that has an impact on family members’ lending or investment decision.

## Conclusion

Family financing has become a powerful channel for entrepreneurs to obtain entrepreneurial funding due to its advantages in transaction costs, borrowing interest, and repayment requirements (Coleman and Robb, 2010). However, some studies have pointed out that household financing will bring entrepreneurs moral burdens or non-financial obligations. It is a substitute for entrepreneurs when they cannot obtain formal funding, such as bank loans (Au and Kwan, 2009; Sieger and Minola, 2017). The literature has emphasized that the motivation of family members to provide financial support to entrepreneurs includes but is not limited to altruism, and there is likely to be expected financial or non-financial returns (Arthurs et al., 2009; Arregle et al., 2015; Lee and Persson, 2016; Sieger and Minola, 2017; Ko and McKelvie, 2018). Therefore, based on the signal theory, we analyze the decision-making process of family members providing financial support to entrepreneurs. At the same time, considering the crucial role of entrepreneurs in startups, pay attention to the signaling effect of entrepreneurs’ positive psychological capital in obtaining family financing (Ensley et al., 2006).

First, as an intent signal, psychological capital can support entrepreneurs to obtain family lending. Because, in the family loan of entrepreneurs, family members tend to consider the vital issue of entrepreneur’s debt performance—moral hazard–thus paying more attention to intent signals, such as entrepreneur’s individual qualities (Jensen and Meckling, 1976; Holmstrom and Tirole, 1997). Entrepreneurs with poor individual quality are more likely to default for family members. Therefore, no-cost signals, such as individual quality in the entrepreneur’s family lending relationship have an essential impact on lending decisions (Stiglitz, 1975; Spence, 1976). Anglin et al. (2018) believe that entrepreneurs with positive psychological capital are trustworthy. Entrepreneurs with positive psychological capital perform well in terms of capacity allocation, risk resolution, goal achievement, and thus promotion of corporate sustainability. Therefore, the opportunistic behavior of entrepreneurs can be effectively avoided, thereby reducing the risk of debt repayment (Karra et al., 2006).

Second, corporate competitive advantage and firm performance have a positive moderating effect on the relationship between psychological capital and family lending raised. Family members as creditors do not directly share the entrepreneurial risk, but the entrepreneurial practice is directly related to the entrepreneur’s capital strength. The promising development prospects and performance of startups means that entrepreneurs’ economic strength is constantly increasing, and they can increase insurance for entrepreneurs to fulfill their debt contracts (Ko and McKelvie, 2018). As a result of entrepreneurial practice, the business performance and industry status of a startup enterprise is an intuitive manifestation of the entrepreneur’s business ability and corporate market position and serves as a quality signal to provide support for family members’ financial decision-making.

## Data Availability Statement

The raw data supporting the conclusions of this article will be made available by the authors, without undue reservation.

## Author Contributions

ES has collected the data. XZ, XS, and ES wrote the first draft. XZ and LZ analyzed the data, contributed to research problem formulation, theory, analysis, and conclusion. All authors contributed to the article and approved the submitted version.

## Conflict of Interest

The authors declare that the research was conducted in the absence of any commercial or financial relationships that could be construed as a potential conflict of interest.

## Publisher’s Note

All claims expressed in this article are solely those of the authors and do not necessarily represent those of their affiliated organizations, or those of the publisher, the editors and the reviewers. Any product that may be evaluated in this article, or claim that may be made by its manufacturer, is not guaranteed or endorsed by the publisher.
